# MSK1 functions as a transcriptional coactivator of p53 in the regulation of *p21* gene expression

**DOI:** 10.1038/s12276-018-0160-8

**Published:** 2018-10-10

**Authors:** Jihye Ahn, Jin Gyeong Lee, Chuevin Chin, Suna In, Aerin Yang, Hee-Sung Park, Jaehoon Kim, Jeong Hyeon Park

**Affiliations:** 1 Department of Biological Sciences, Korea Advanced Institute of Science and Technology, Daejeon, 34141 South Korea; 2 Institute of Fundamental Sciences, Massey University, Palmerston North, 4410 New Zealand; 3 Department of Chemistry, Korea Advanced Institute of Science and Technology, Daejeon, 34141 South Korea

## Abstract

Mitogen- and stress-activated kinase 1 (MSK1) is a chromatin kinase that facilitates activator-dependent transcription by altering chromatin structure through histone H3 phosphorylation. The kinase activity of MSK1 is activated by intramolecular autophosphorylation, which is initially triggered by the activation of upstream mitogen-activated protein kinases (MAPKs), such as p38 and ERK1/2. MSK1 has been implicated in the expression of *p21*, a p53 target gene; however, the precise connection between MSK1 and p53 has not been clearly elucidated. Here, using in vitro and cell-based transcription assays, we show that MSK1 functions as a transcriptional coactivator of p53 in *p21* expression, an action associated with MAPK-dependent phosphorylation of MSK1 and elevated kinase activity. Of special significance, we show that MSK1 directly interacts with p53 and is recruited to the *p21* promoter, where it phosphorylates histone H3 in a p53-dependent manner. In addition, phosphomimetic mutant analysis demonstrated that negative charges in the hydrophobic motif are critical for serine 212 phosphorylation in the N-terminal kinase domain, which renders MSK1 competent for histone kinase activity. These studies suggest that MSK1 acts through a direct interaction with p53 to function as a transcriptional coactivator and that MSK1 activation by upstream MAPK signaling is important for efficient *p21* gene expression.

## Tumor suppression: another link in a signaling chain

New drugs to fight tumors could be developed, thanks to research revealing how a protein called MSK1 interacts with and assists another protein, “p53,” which has well-established tumor-suppressing activity. Researchers led by Jeong Hyeon Park at Massey University in New Zealand and Jaehoon Kim at the Korea Advanced Institute of Science and Technology in South Korea studied the function of the MSK1 protein in cultured human cells. They uncovered details of a previously unknown direct interaction between the MSK1 and p53 proteins. This allows the MSK1-p53 combination to increase the activity of a gene that codes for another protein, p21. The p21 protein then participates in further molecular processes that can restrict cell growth and multiplication. Drugs that control MSK1 activity might offer new opportunities to combat the uncontrolled multiplication of cancer cells.

## Introduction

Activation of mitogen-activated protein kinases (MAPKs) by multiple upstream kinase cascades transduces extracellular signals that regulate diverse cellular processes, including gene expression. Mitogen- and stress-activated kinases 1 and 2 (MSK1/2) are 2 of 11 downstream kinases known to be regulated by MAPKs^1^. MSK1/2 contain two independent kinase domains, an N-terminal kinase domain (NKD) and a C-terminal kinase domain (CKD), connected by a regulatory linker region. Two canonical MAPKs, p38 and ERK1/2, activate MSK1/2 by interacting with the MAPK-binding domain near the C-terminal nuclear localization sequence within MSK1/2^1,2^.

MSK1/2 are unique among MAPK-activated protein kinases in that the CKD is first activated by MAPK-mediated phosphorylation, which subsequently activates the NKD through intramolecular autophosphorylation^1,3^. Mutagenesis studies of MSK1 suggest that upstream MAPKs phosphorylate three critical serine/threonine residues located in the linker region (serine 360), the activation loop of the CKD (threonine 581), and near a putative autoinhibitory domain (threonine 700)^4,5^. Activated CKD autophosphorylates the activation loop in NKD (serine 212) and the hydrophobic motif in the linker region (serine 376/381). The activated NKD phosphorylates target proteins and additional residues in the C terminus of MSK1 (serine 750/752/758).

MSK1/2 double-knockout mice have no apparent phenotype under specific pathogen-free conditions but display hypersensitive immune responses to stimulation with lipopolysaccharide, owing to a defect in anti-inflammatory cytokine gene expression^6^. Transcriptional regulation by MSK1 has been reported to involve phosphorylation of transcription factors and nucleosomes^3^. On the one hand, MSK1-mediated phosphorylation of transcription factors, such as CREB (cAMP-response element-binding protein), ATF1, nuclear factor-κB (NF-κB), and RARα, increases protein stability or transcriptional activity^7–10^. On the other, MSK1 also has a role in the nucleosomal response by phosphorylating the chromatin components histone H3 and HMGN1 upon MAPK signaling^11,12^. The phosphorylation of histone H3 at serine 10 and serine 28 (hereafter, H3S10 and H3S28) in the promoter region is one of the earliest epigenetic modifications during gene activation^13–15^. Although MSK1-mediated histone phosphorylation correlates well with transcription from target gene promoters, the molecular mechanisms by which histone phosphorylation enhances transcription are not clearly understood^3,8,11,16,17^.

One of the target genes regulated by MSK1 is *p21*, which encodes a cyclin-dependent kinase inhibitor. *p21* is also a well-established p53 target gene that has a critical role in p53-mediated tumor suppressive functions by inducing growth arrest and cellular senescence^18,19^. Various histone deacetylase (HDAC) inhibitors have been shown to activate *p21* expression through regulation of histone acetylation of proximal promoter elements, such as Sp1-binding sites^20–22^. More importantly, MSK1 appears to mediate phosphorylation of histone H3 in the promoter region, an action that is indispensable for the activation of *p21* by HDAC inhibitors, suggesting that p53 and MAPK-MSK1 pathways act synergistically during transcriptional activation of *p21*^23^. However, given that multiple transcriptional activators are involved in regulating *p21* gene expression^24^, the precise molecular mechanisms by which MSK1 is targeted to the *p21* promoter and regulates *p21* gene expression remain to be elucidated.

Here we demonstrate that p53 enhances MSK1-mediated phosphorylation of histone H3 on chromatin templates through direct interaction with MSK1 and thereby upregulates *p21* transcription. We also employed a DNA fragment shuffling method to generate multiple combinations of phosphomimetic mutations on MSK1 to correlate its kinase activity-dependent coactivator function with *p21* expression. Our results suggest that dynamic posttranslational modifications of MSK1 and enhanced MSK1 kinase activity are important for the coactivator function of MSK1 in the p53-dependent transcription of *p21*.

## Materials and methods

### Cell culture and ectopic gene expression

Human cell lines were maintained in Glutamax-DMEM (Invitrogen) supplemented with 10% (v/v) fetal bovine serum (FBS) (Sigma), 50 U/ml penicillin, 50 μg/ml streptomycin, and 0.125 µg/ml amphotericin B (Sigma) at 37 °C in a 5% CO_2_ humidified incubator. Sf9 cells were cultured in Grace’s insect medium (Gibco-Invitrogen) supplemented with 10% FBS, 0.1% pluronic acid (Sigma), and 10 µg/ml gentamicin (Sigma) in normal atmospheric conditions. Baculovirus was generated according to the manufacturer’s instructions (Gibco-Invitrogen).

### Recombinant protein preparations

Recombinant GST-MSK1 protein was purchased from Invitrogen. The cDNAs expressing MSK1 and a constitutively active MKK6 mutant (MKK6ca) were obtained from Dr Aya Fukuda (University of Tsukuba). Expression and purification of epitope-tagged transcription factors and MSK1 using bacterial, baculovirus, and human cell expression systems were performed as previously described^25^. Recombinant SBP-MSK1 expressed from the pNTAP vector was purified using streptavidin resin as described in the manufacturer’s manual (Agilent Technologies).

### Recombinant chromatin assembly and in vitro kinase assay

To obtain physiologically spaced nucleosome particles on a chromatin fiber, the ideal histone to DNA mass ratio was determined and used to assemble recombinant chromatin as described previously^26,27^. In vitro kinase assays were performed in a reaction containing 150 ng of substrate (chromatin or histone octamer), 50 ng of transcription activator (p53, Sp1, or RAR-RXR), and 50 ng of MSK1, supplemented with 10 mM HEPES (pH 7.4), 10 mM MgCl_2_, 50 mM NaCl, 10 mM MnCl_2_, and 2 mM ATP. After incubation at 30 °C for 30 min, the reactions were resolved by gradient SDS-polyacrylamide gel electrophoresis (8~ 15%) and subjected to immunoblotting.

### Protein interaction assay

For glutathione *S*-transferase (GST)-pull-down assays, purified GST or GST-p53 immobilized on glutathione-Sepharose 4B beads (GE Healthcare) was incubated with purified FLAG-MSK1 in binding buffer [20 mM Tris-Cl (pH 7.5), 200 mM KCl, 0.2 mM EDTA, 20% glycerol, 0.1% NP-40, 0.5 mg/ml bovine serum albumin (BSA), and 0.5 mM phenylmethylsulfonyl fluoride (PMSF)] at 4 °C for 3 h, and then the beads were extensively washed with binding buffer without BSA. Bound proteins were analyzed via immunoblotting with anti-MSK1 antibody. For co-immunoprecipitation assays, HEK293T cells were transfected with combinations of FLAG-MSK1 and HA-p53 expression plasmids using iN-fect™ transfection reagent (iNtRON Biotechnology). After 48 h, cell lysates prepared using lysis buffer [20 mM Tris-Cl (pH 7.5), 300 mM KCl, 0.2 mM EDTA, 0.1% NP-40, 20% glycerol, and 0.2 mM PMSF] were incubated with M2 agarose (Sigma), and then the beads were extensively washed with lysis buffer. Bound proteins were analyzed by immunoblotting.

### In vitro transcription assay

An in vitro transcription assay of a chromatinized p53ML array template was performed as follows: chromatin template (containing 40 ng DNA) was incubated with p53 (10 ng) in 0.5 × HAT buffer [10 mM HEPES (pH 7.8), 30 mM KCl, 2.5 mM dithiothreitol (DTT), 0.25 mM EDTA, and 5 mM sodium butyrate] at 30 °C for 20 min in a 20 μl reaction and then further incubated with p300 (15 ng), 20 μM acetyl-CoA, and the indicated amounts of purified MSK1 at 30 °C for 30 min in a 25 μl reaction. Preinitiation complex formation was initiated by adding 5 μl of HeLa cell-derived nuclear extract at room temperature for 20 min. Transcription was carried out at 30 °C for 40 min in a final volume of 50 μl containing 25 mM HEPES (pH 8.2), 11–16% glycerol, 4 mM MgCl_2_, 60 mM KCl, 5 mM DTT, 0.5 mM ATP and CTP, 25 μM UTP, 0.1 mM 3′-*O*-methyl-GTP, 10 μCi [α-^32^P] UTP (10 μCi/μl, 3000 Ci/mmol), 0.5 mg/ml BSA, and 10 U RNasin (Promega). The reaction was stopped by the addition of 150 μl of stop buffer [150 mM sodium acetate (pH 5.2), 0.5% SDS, and 10 mM EDTA] and further incubated at 37 °C for 30 min with 30 μg of proteinase K (Sigma). Radiolabeled RNA was extracted with phenol/chloroform/isoamyl alcohol (25:24:1), precipitated by ethanol, resolved on a 5% polyacrylamide gel (19:1) containing 8 M urea, and analyzed by autoradiography.

### Quantitative reverse transcription-PCR (RT-qPCR)

A QuantiTect primer assay (Qiagen) was used for real-time PCR assays with a Quantifest SYBR Green RT-PCR kit [*p21* (QT00062090) and GUSB (QT00046046)]. Triplicate samples of 4 μl from a total RNA preparation were analyzed by adding 21 μl of a combined reverse transcriptase and PCR reaction mixture (0.2 μM forward and reverse primers, 12.5 μl of 2 × SYBR reaction mixture, 0.2 μl of reverse transcriptase, and Taq DNA polymerase mixture). cDNA synthesis was started by incubation at 45 °C for 30 min and followed by conventional quantitative PCR (95 °C for 5 s and 60 °C for 30 s, 40 cycles) in an LC480 real-time PCR machine (Roche).

### DNA cloning and golden gate shuffling mutagenesis

To generate MSK1 phosphomimetic mutants, MSK1 cDNA was first divided into five smaller fragments by PCR reactions using sequence-specific oligonucleotide primers (Supplementary Table 1). The *Bsa*l recognition sequence was added to the 5′-end of forward and reverse primers so that each MSK1 fragment was flanked by a *Bsa*l recognition site at both the 5′- and 3′-ends. These fragments were then subcloned individually into a modified pUC19 vector, pICH41021 (kindly provided by Dr Kee Hoon Sohn at POSTECH), generating five pICH-MSK1 entry vectors containing cDNA fragments corresponding to the amino acid residues 1–227, 228–428, 429–690, 691–792, and 793–802 of MSK1. Selected serine/threonine residues in each fragment were mutated to glutamic acid with a PCR-based site-directed mutagenesis protocol using a KAPA HiFi^TM^ PCR kit (Kapa Biosystems) and primer pairs for introduction of desired mutations (Supplementary Table 2). The pNTAP-*Bsa*l-LacZ recipient vector is a modified pNTAP-A expression vector (Stratagene) constructed for this study that contains a LacZ cassette whose 5′- and 3′-ends are flanked by *Bsa*l restriction sites in inverse orientation. Assembly of full-length MSK1 into the pNTAP-*Bsa*l-LacZ recipient vector was conducted in a single step^28^ by mixing the following components together in a single reaction: 100 ng of pNTAP-*Bsa*l-LacZ destination vector, 120 ng of each pICH donor vector containing wild-type or mutant MSK1 cDNA fragments, 1 µl of *Bsa*l enzyme (NEB), 2 µl of 10 × T4 ligase buffer (NEB), 2 µl of 10 × BSA (1 mg/ml), and 1 µl of T4 ligase (NEB) in a total volume of 20 µl. After completion of the assembly, *Escherichia coli* was transformed with 10 µl of the reaction and plated on Luria-Bertani plates supplemented with 50 μg/ml kanamycin and X-gal (5-bromo-4-chloro-3-indolyl-β-d-galactopyranoside). The plasmids expressed in white colonies were purified and analyzed by restriction enzyme digestion and DNA sequencing.

### Chromatin immunoprecipitation assay

HCT116 cells were treated with 0.5 μM doxorubicin (Sigma) for the indicated times. The procedures for chromatin immunoprecipitation (ChIP) analysis and primers for quantitative PCR have been described previously^29^.

### Antibodies

The following antibodies were obtained commercially: anti-p53 (Santa Cruz Biotechnology); anti-p21, anti-MSK1, anti-MSK1 T581-P and anti-H3S10-P (Abcam); and anti-MSK1 S212-P (R&D Systems). All other antibodies were purchased from Sigma.

## Results

### MSK1 phosphorylates histone H3 within a chromatinized *p21* promoter in a p53-dependent manner

**Fig. 1:**
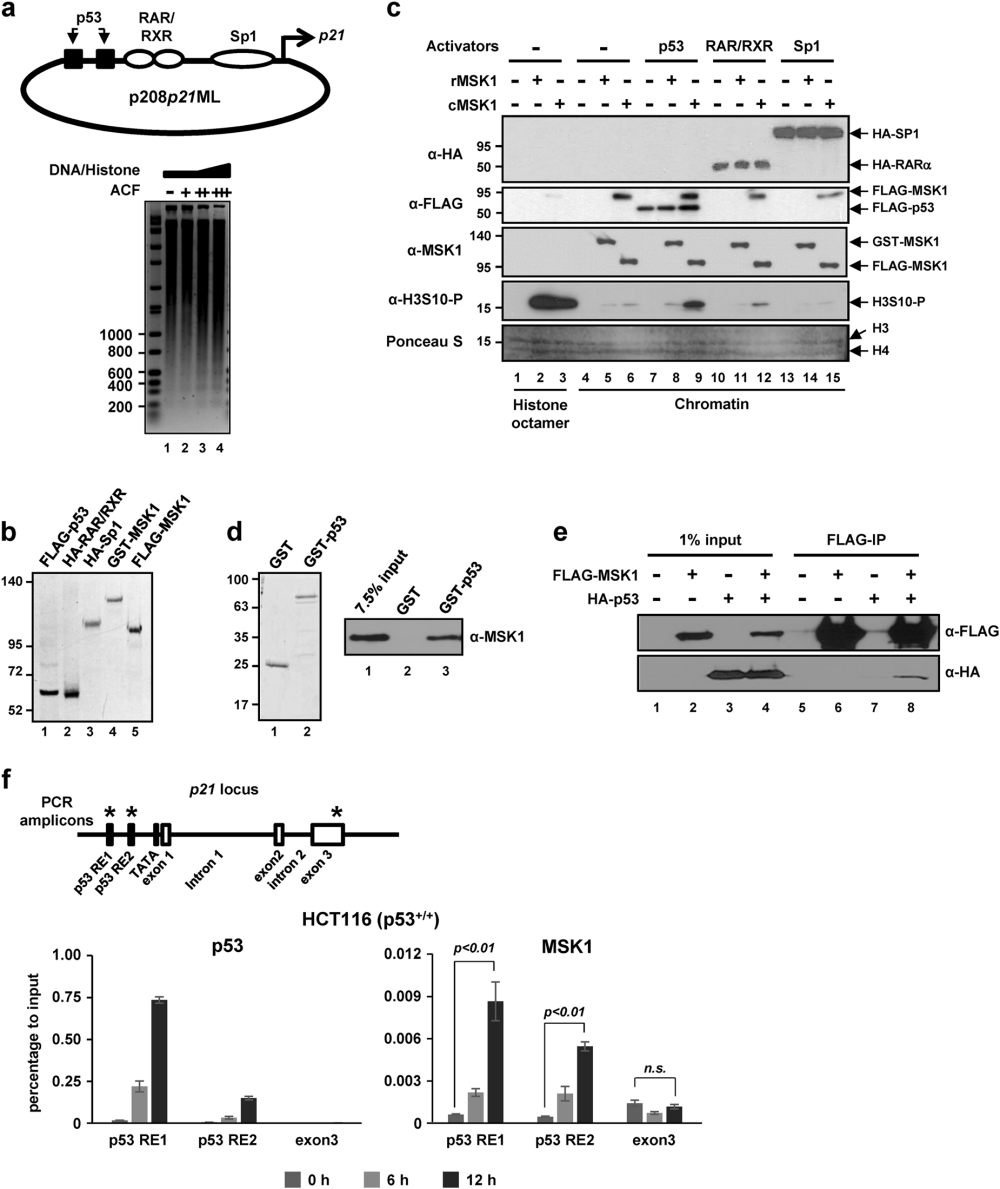
p53-dependent chromatin phosphorylation by MSK1 in vitro. **a** The binding sites of three transcription factors and the transcription start site are indicated in the 2.4 kb of the *p21* promoter region (top panel). Recombinant chromatin was assembled with a *p21* promoter-containing plasmid (p208*p21*ML) and purified core histones using an ACF/NAP1 chromatin assembly system. Micrococcal nuclease digestion and agarose gel electrophoresis analyses of the assembled chromatins with different ratios of DNA to histone were performed (bottom panel). **b** SDS-PAGE/Coomassie blue staining of purified transcription factors and MSK1. **c** Reactions containing substrate (histone octamer or chromatin), a transcription activator, and MSK1 (rMSK1, recombinant MSK1; cMSK1, cellular MSK1 activated by MKK6ca co-expression), where indicated, were subjected to in vitro kinase assays. For histone octamer and chromatin substrates, 5 ng and 50 ng, respectively, of MSK1 were added. The protein components and the level of H3S10 phosphorylation in each reaction were examined by immunoblotting with the indicated antibodies. The total histone amounts are shown by protein staining of the membrane. **d** SDS-PAGE/Coomassie blue staining of purified GST and GST-p53 (left panel). Binding of FLAG-MSK1 to GST-p53 vs. GST was monitored by immunoblotting with anti-MSK1 antibody (right panel). **e** Intracellular association of p53 and MSK1. HEK293T cells were transfected with plasmids expressing FLAG-MSK1 and HA-p53 as indicated. Cell extracts were incubated with M2 agarose, and the bound proteins were scored by immunoblotting with the indicated antibodies. **f** ChIP analyses of p53 and MSK1 on the *p21* locus during p53-dependent transcription. A schematic representation of the *p21* locus with the three amplicons (indicated by asterisks) used for ChIP-qPCR (top panel). HCT116 colorectal cancer cells expressing p53 (p53^+/+^) were treated with doxorubicin for the indicated times and ChIP analyses were performed with the indicated antibodies (bottom panels). Error bars indicate the SDs from three independent ChIP analyses. ChIP analysis with IgG showed negligible signals (data not shown). The statistical significance of the MSK1 recruitment upon doxorubicin treatment was evaluated by Student’s *t*-test. RE responsive element

MSK1-dependent histone phosphorylation occurs in the *p21* promoter region during transcriptional activation^23^, but how MSK1 is targeted to the *p21* promoter during this process is not well understood. We hypothesized that MSK1 is recruited to the promoter region of *p21* by interaction with specific transcription factors. As MSK1 cannot phosphorylate chromatin without DNA-binding transcriptional activators^24^, a chromatinized *p21* promoter was used as a substrate to identify a transcriptional activator for MSK1 targeting. The p208*p21*ML plasmid containing 2.4 kb of the *p21* promoter region was assembled into a nucleosomal array using an ACF-NAP1 chromatin assembly system (Fig. 1a, top). Micrococcal nuclease digestion produced regularly spaced 200 bp ladders (Fig. 1a, bottom, lane 4), indicating that the assembled recombinant chromatin mimics physiological nucleosomes and is therefore suitable for chromatin kinase assays.

To reconstitute activator-dependent chromatin phosphorylation, we chose three transcriptional activators, p53, RAR/RXR heterodimer, and Sp1, as potential targeting activators (Fig. 1a). Recombinant FLAG-p53 (expressed in *E. coli*), HA-RAR/RXR (expressed in Sf9 cells), and HA-Sp1 (expressed in HEK293T cells) proteins were prepared via antibody-based affinity purification (Fig. 1b)^10,23,30,31^. In addition, we employed recombinant MSK1 (rMSK1) obtained from a commercial source as a GST-tagged protein (GST-MSK1) expressed in Sf9 cells and cellular MSK1 (cMSK1) purified from HEK293T cells co-expressing FLAG-MSK1 and a p38 pathway-activating MKK6 mutant (MKK6ca) (Fig. 1b).

We then assessed the kinase activity of MSK1 on chromatin templates using an in vitro histone kinase assay by measuring H3S10 phosphorylation status (Fig. 1c). In the absence of activator, MSK1 preparations from both Sf9 cells (rMSK1) and HEK293T cells (cMSK1) showed strong phosphorylation activity toward free histone H3 but not toward the chromatin substrate (Fig. 1c, anti-H3S10-P immunoblot, lanes 2 and 3 vs. lanes 5 and 6). Importantly, among the three transcriptional activators tested, only p53 induced a significant degree of cMSK1-mediated H3S10 phosphorylation of the chromatin template (Fig. 1c, anti-H3S10-P immunoblot, lane 9 vs. lanes 12 and 15). Interestingly, rMSK1 exhibited strong kinase activity toward free histone substrates but failed to phosphorylate chromatin, even in the presence of p53 (Fig. 1c, anti-H3S10-P immunoblot, lane 8). These results indicate that p53 could be a principal transcriptional activator that recruits MSK1 for histone phosphorylation around the *p21* promoter region, a function that may also require upstream MAPK activation signals that lead to phosphorylation of MSK1 at a specific site.

To assess the possibility that p53-dependent H3S10 phosphorylation reflects direct binding of MSK1 to p53, we tested interactions of purified FLAG-MSK1 and GST-p53 proteins (Fig. 1d, left). Specific enrichment of MSK1 in GST-p53 pull-down experiments confirmed a direct interaction of p53 with MSK1 in vitro (Fig. 1d, right). In a test for intracellular interactions, ectopically expressed HA-p53 protein was co-immunoprecipitated by an anti-FLAG antibody only in the presence of FLAG-MSK1 (Fig. 1e). Taken together, our results imply that MSK1 directly binds to p53 in vitro and in cells.

Next, we examined whether MSK1 is recruited to the promoter region of p53 target genes in a p53-dependent manner. To this end, we used p53-dependent induction of *p21* by doxorubicin treatment to demonstrate genomic site-specific accumulation of p53 and MSK1 in HCT116 (p53^+/+^) cells expressing wild-type p53. Three regions of the *p21* locus were probed for relative enrichment of p53 and MSK1 by ChIP assays using primers that amplified the corresponding regions (Fig. 1f, top). These analyses showed that doxorubicin treatment induced a simultaneous increase in p53 and MSK1 occupancy at p53-responsive elements but not at the exon 3 region (Fig. 1f, bottom). In contrast, MSK1 recruitment to the same genomic sites remained at a basal level during the course of doxorubicin treatment in p53-null isogenic HCT116 (p53^−/−^) cells (Supplementary Fig. 1). These results suggest that p53 and MSK1 are recruited to the same genomic locus of the *p21* gene upon p53 activation.

### MSK1 enhances p53-dependent *p21* transcription in cells and in vitro

**Fig. 2:**
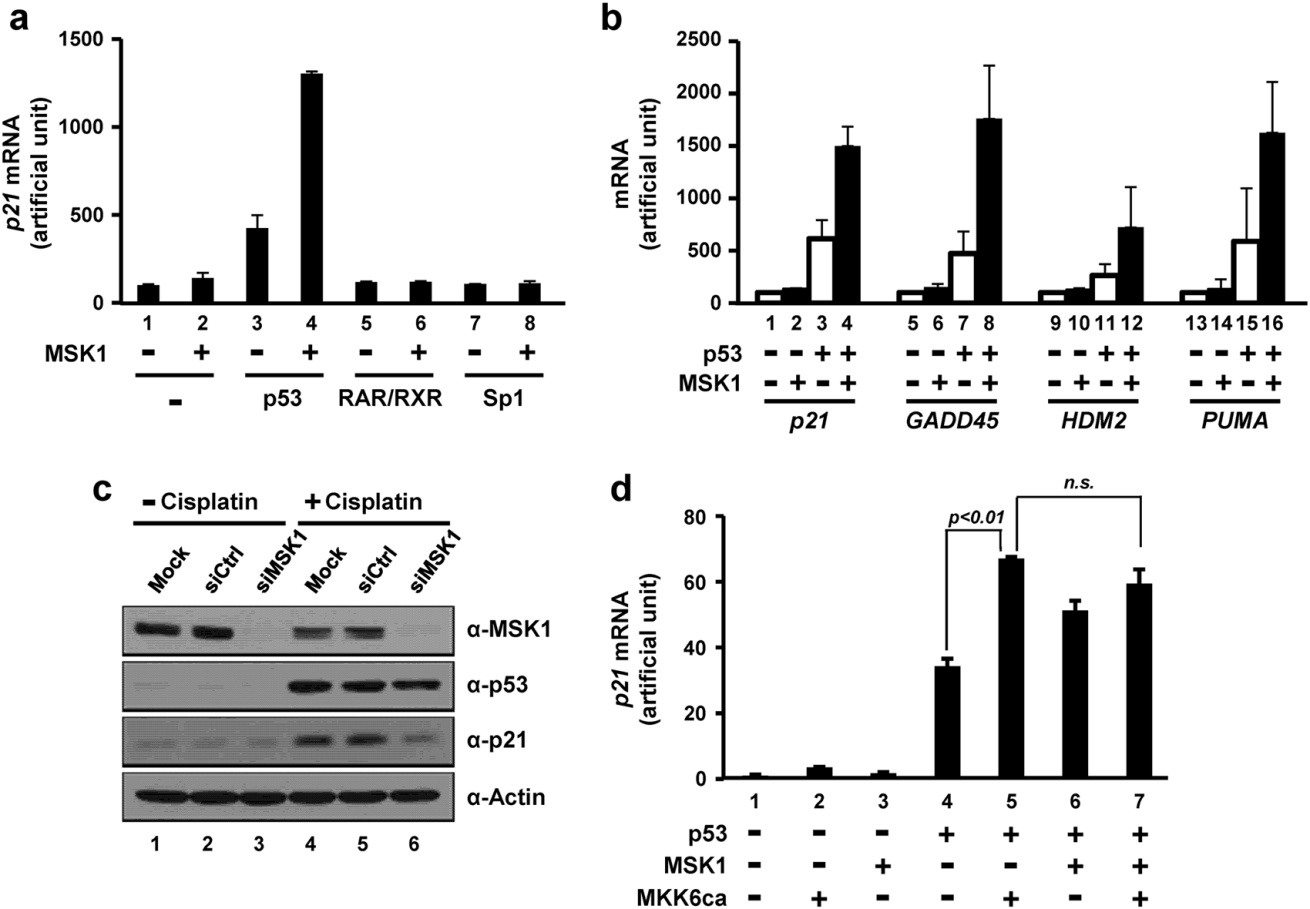
MSK1 functions as a transcriptional coactivator of p53 in cells. **a** Synergistic effects of p53 and MSK1 in endogenous *p21* transcription. p53-null H1299 cells were transfected with a combination of plasmids encoding transcription activators and MSK1. Cells for ectopic RAR/RXR expression were treated with 9-*cis*-retinoic acid. **b** General coactivator functions of MSK1 in p53 target gene expression. H1299 cells were transfected with p53 and MSK1 expression plasmids as indicated. **c** Requirement of endogenous MSK1 for p21 expression. MCF-7 cells pretreated with nonspecific (siCtrl) and MSK1 (siMSK1) siRNA, as indicated, were treated with 30 μM cisplatin. Cell extracts were subjected to immunoblotting analysis with the indicated antibodies. **d** Activation of the MSK1 pathway enhances endogenous *p21* transcription. H1299 cells were transfected with vectors encoding a combination of p53, MSK1, and a constitutively active MKK6 mutant (MKK6ca) as indicated. **a**, **b**, and **d** Endogenous mRNA levels were measured via one step reverse-transcriptase (RT)-coupled qPCR and normalized relative to *β-glucuronidase* expression. Error bars represent the mean SD of triplicate samples. The significance of the differences in *p21* expression (**d**) was evaluated using Student’s *t*-test. Comparable levels of ectopic protein expression were observed in all the examined samples (data not shown)

To test the coactivator function of MSK1 in cells, we analyzed endogenous *p21* mRNA expression in the presence of ectopically expressed MSK1 and p53. p53-null H1299 cells were transfected with plasmids that express MSK1 and one of the transcriptional activators, p53, RAR/RXR, or Sp1, after which *p21* mRNA levels were examined by quantitative PCR analyses (Fig. 2a). *p21* expression was not responsive to overexpression of either RAR-RXR or Sp1 (Fig. 2a, lanes 5 and 7). In addition, MSK1 by itself and in combination with RAR-RXR or Sp1 did not result in a significant increase in *p21* mRNA (Fig. 2a, lanes 2, 6, and 8). However, ectopic expression of MSK1 and p53 enhanced *p21* transcription up to threefold relative to p53 alone (Fig. 2a, lane 4 vs. lane 3), suggesting a coactivator function of MSK1 in p53-mediated *p21* transcription.

To determine whether the coactivator function of MSK1 also occurs with other p53 target genes, we analyzed the transcriptional responses of the p53 targets *GADD45*, *HDM2*, and *PUMA* in H1299 cells (Fig. 2b). We found that MSK1 enhanced p53-dependent transcription of all the tested genes by two- to threefold, demonstrating a general coactivator function of MSK1 in p53 target gene expression.

To test the role of endogenous MSK1 in p53-dependent *p21* expression, we examined cisplatin-induced, p53-dependent p21 expression in cells depleted of MSK1 through treatment with small interfering RNA targeting MSK1 (Fig. 2c). We observed clear concomitant increases in p21 upon p53 activation by cisplatin treatment (Fig. 2c, anti-p21 and anti-p53 immunoblots, lanes 4 and 5 vs. lanes 1 and 2), an effect that was abolished by depletion of endogenous MSK1 (Fig. 2c, anti-p21 immunoblot, lane 6 vs. lane 5). These data confirm a transcriptional coactivator function of MSK1 in p53 target gene expression.

Given that ectopic MSK1 expression without additional upstream activation signals enhanced p53-dependent transcription (Fig. 2a, b), we examined whether an upstream kinase that activates MSK1 produces a synergistic effect on p53-dependent transcription. To this end, we employed MKK6ca, a constitutively active MKK6 mutant that has been widely used to specifically activate p38^32–35^. Consistent with the role of p38 in enhancing p53-dependent transcription^36–38^, ectopic expression of MKK6ca enhanced p53-dependent *p21* transcription (Fig. 2d, lane 5 vs. lane 4). However, simultaneous overexpression of MKK6ca and MSK1 did not further increase p53-dependent *p21* transcription compared with MKK6ca alone (Fig. 2d, lane 7 vs. lane 5), suggesting that endogenous MSK1 is sufficient to fully recapitulate the stimulatory effect of the MKK6ca-p38-MSK1 pathway on p53-dependent *p21* transcription.

**Fig. 3:**
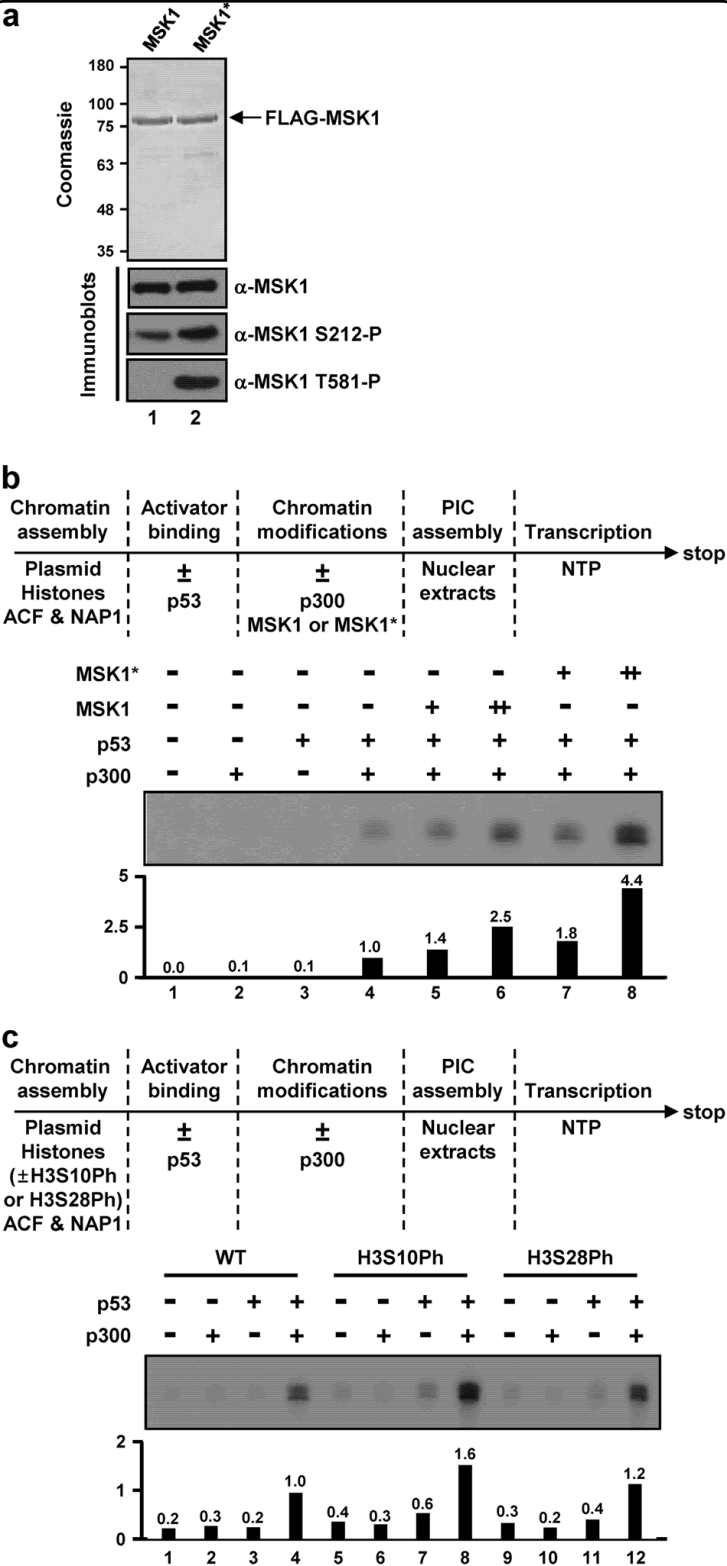
Coactivator function of MSK1 in p53-mediated in vitro transcription. **a** SDS-PAGE/Coomassie blue staining and immunoblot analyses with the indicated antibodies for purified FLAG-MSK1 from HEK293T cells in the absence (MSK1, lane 1) and presence (MSK1*, lane 2) of MKK6ca co-expression. **b** Effects of MSK1 in p53-mediated in vitro transcription of a chromatin template. Standard p53- and p300-dependent transcription from a chromatinized p53ML plasmid was performed according to the scheme in the top panel. Reactions contained 5 ng (lanes 5 and 7) or 20 ng (lanes 6 and 8) of MSK1 where indicated. **c** Direct effects of histone H3 phosphorylation in the p53-mediated in vitro transcription of a chromatin template. In vitro transcription assays were performed with chromatin templates assembled with recombinant histone octamers containing fully phosphorylated H3S10 or H3S28. Relative transcription levels were measured using a phosphoimager and normalized to that of p53 and p300 addition (**b** and **c**, lane 4)

To further address the contribution of MSK1 activation to p53 target gene transcription in a nucleosomal context, we employed an in vitro transcription assay using a chromatinized G-less cassette template containing p53-binding sites^29^. rMSK1 was prepared from HEK293T cells in the presence (MSK1*) and absence (MSK1) of MKK6ca co-expression. As expected, the presence of an MKK6ca-driven activation signal in the cell induced hyperphosphorylation of MSK1 in the activation loop of NKD (serine 212) and CKD (threonine 581) (Fig. 3a). The transcriptional coactivator function of purified MSK1 was assessed using in vitro transcription assays, as shown schematically in Fig. 3b (top panel). Compared with the basal transcriptional activation observed with p53 and p300 (Fig. 3b, lane 4), inclusion of MSK1 increased transcription up to 2.5-fold (Fig. 3b, lanes 5 and 6 vs. lane 4). More importantly, MKK6ca-activated MSK1* resulted in further transcription enhancement (up to 4.4-fold) relative to basal transcription (Fig. 3b, lanes 7 and 8 vs. lane 4). These results suggest that upstream kinase-mediated activation of MSK1 enhances MSK1 transcriptional coactivator activity in p53-dependent transcription.

To more directly assess the effects of MSK1-mediated histone phosphorylation on chromatin transcription, we generated histone octamers containing homogenously phosphorylated H3S10 or H3S28 using an amber suppression method^39^ and assembled them into chromatin for p53-dependent transcription assays (Fig. 3c). Chromatin templates assembled with phosphorylated H3S10 and H3S28 exhibited 1.6- and 1.2-fold increases, respectively, in p53- and p300-dependent transcription compared with that of unmodified histone H3 (Fig. 3c, lanes 8 and 12 vs. lane 4). These results demonstrate a direct stimulatory effect of histone H3 phosphorylation on p53-dependent transcription, as well as a more significant contribution of H3S10 phosphorylation relative to H3S28 phosphorylation. Collectively, these results suggest that concomitant activation of p53 and MSK1 has an important role in p53 target gene expression, possibly involving cooperative interactions of p53 and MSK1 to achieve an adequate nucleosomal response.

### Negative charges on serine 376/381 residues in the hydrophobic motif of the linker region constitutively activate MSK1

**Fig. 4:**
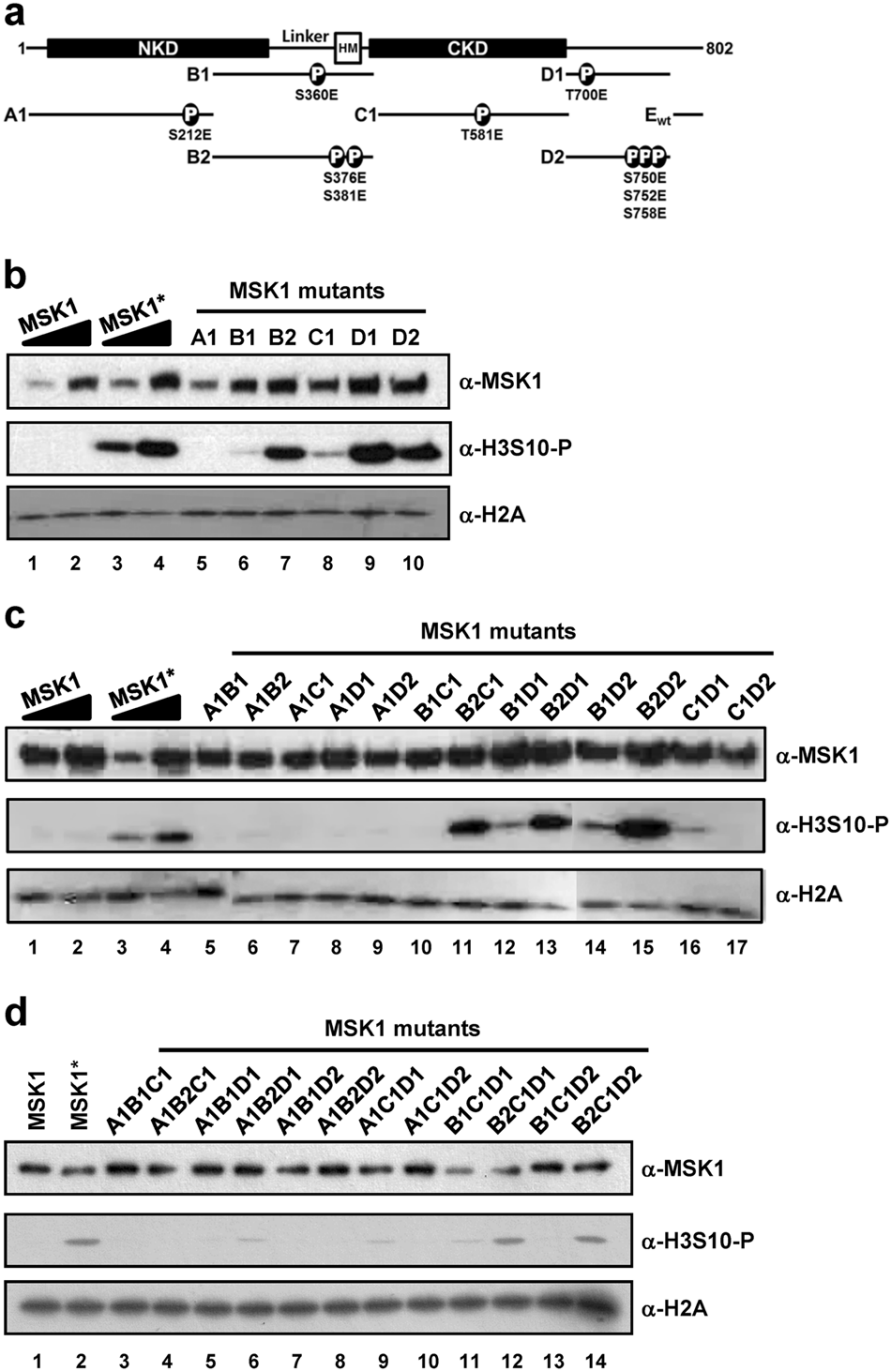
Characterization of histone phosphorylation activities of MSK1 phosphomimetic mutants. **a** Schematic diagram of MSK1 with the N-terminal kinase domain (NKD), hydrophobic motif (HM) in the linker region and C-terminal kinase domain (CKD). Phosphomimetic mutations are indicated in the A1 (S212E), B1 (S360E), B2 (S367E and S381E), C1 (T581E), and D1 (T700E) and D2 (S750E, S752E, and S758E) fragments. **b**–**d** Reactions containing core histone octamers and purified MSK1 with a single (**b**), double (**c**), and triple (**d**) number of mutant fragments were subjected to in vitro kinase assays. The kinase activities of purified MSK1 were measured by immunoblot with anti-phosphorylated H3S10 antibody. MSK1 and MSK1* wild-type MSK1 were prepared in the absence and presence of MKK6ca co-expression, respectively. The data are representative of at least three independent experiments

It is known that MSK1 is activated through multiple phosphorylation events at serine 360, threonine 581, and threonine 700 mediated by p38 or ERK1/2 MAPKs^4,5^. Previous studies have investigated the kinase activity of MSK1 using only artificial peptide substrates^5^. However, whether specific MSK1 phosphorylation events are correlated with enhanced activity toward genuine target substrates, such as histone H3, has not been clearly demonstrated. To identify specific phosphorylation events that confer constitutive hyperactivity in the absence of upstream activation signals (e.g., MKK6 and p38 MAPK), we introduced phosphomimetic mutations into various sites in MSK1 using the type II restriction enzyme-based DNA shuffling method (Supplementary Fig. 2)^40^. Using this strategy, we successfully generated 35 MSK1 variants containing shuffled fragments of either wild-type MSK1 or MSK1 with phosphomimetic mutations (Fig. 4a, Supplementary Fig. 2).

Expression plasmids for an MSK1 mutant containing an N-terminal SBP (streptavidin-binding peptide)-tag were introduced into HEK293T cells (in the absence of MKK6ca expression) and then individual MSK1 mutants were purified under native conditions using streptavidin-conjugated beads and biotin elution. To directly assess the effects of phosphomimetic mutations on the kinase activity of MSK1, we performed in vitro histone kinase assays by examining H3S10 phosphorylation status.

Phosphomimetic MSK1 mutants containing glutamic acid substitutions at serines 376 and 381 (B2 fragment), threonine 700 (D1 fragment), and serines 750, 752, and 758 (D2 fragment) exhibited comparable levels of H3S10 phosphorylation activity relative to that of MKK6ca-activated MSK1 (Fig. 4b, lanes 7, 9, and 10 vs. lanes 3 and 4), indicating that negative charges at these sites are able to activate the histone kinase activity of the NKD of MSK1. However, MSK1 mutants containing a glutamic acid substitution at serine 212 (A1 fragment), serine 360 (B1 fragment), or threonine 581 (C1 fragment) failed to show histone kinase activity (Fig. 4b, lanes 5, 6, and 8), indicating that negative charges at these sites are not able to mimic the effect of natural phosphorylation events. Importantly, an S212E mutation in the activation loop of the NKD appeared to completely inactivate the kinase activity of the NKD, preventing additional phosphomimetic mutations at other phosphorylation sites from recovering the histone kinase activity (Fig. 4c, lanes 5–9, Fig. 4d, lanes 3–10, Supplementary Fig. 3). Similarly, a T581E mutation in the activation loop of the CKD appeared to inactivate the C-terminal kinase activity, as evidenced by the observation that the stimulatory effect of T700E or S750E/S752E/S758E mutations (Fig. 4b, lanes 9 and 10) was abolished by the additional T581E mutation (Fig. 4c, lanes 16 and 17).

Importantly, we found that the T581E mutation did not block activation of the NKD by S376E/S381E mutations (Fig. 4c, lane 11), suggesting that phosphorylation of the hydrophobic motif in the linker region directly activates the histone kinase activity of the NKD in a CKD-independent manner. Consistent with this, most MSK1 variants containing phosphomimetic mutations in the hydrophobic motif (i.e., B2 fragment-containing MSK1 mutants) exhibited enhanced H3S10 phosphorylation activity, as shown in Fig. 4b–d and summarized in Supplementary Fig. 3. Thus, introduction of negative charges in the hydrophobic motif (S376/381E) constitutively activates the histone kinase activity of NKD, even if the CKD is inactivated by a T581E mutation. These results suggest that phosphorylation of the hydrophobic motif in the linker region functions as the prime signal transducer that directly turns on the histone kinase activity of MSK1.

### Characterization of the phosphorylation status of activation loops in MSK1 phosphomimetic mutants

**Fig. 5:**
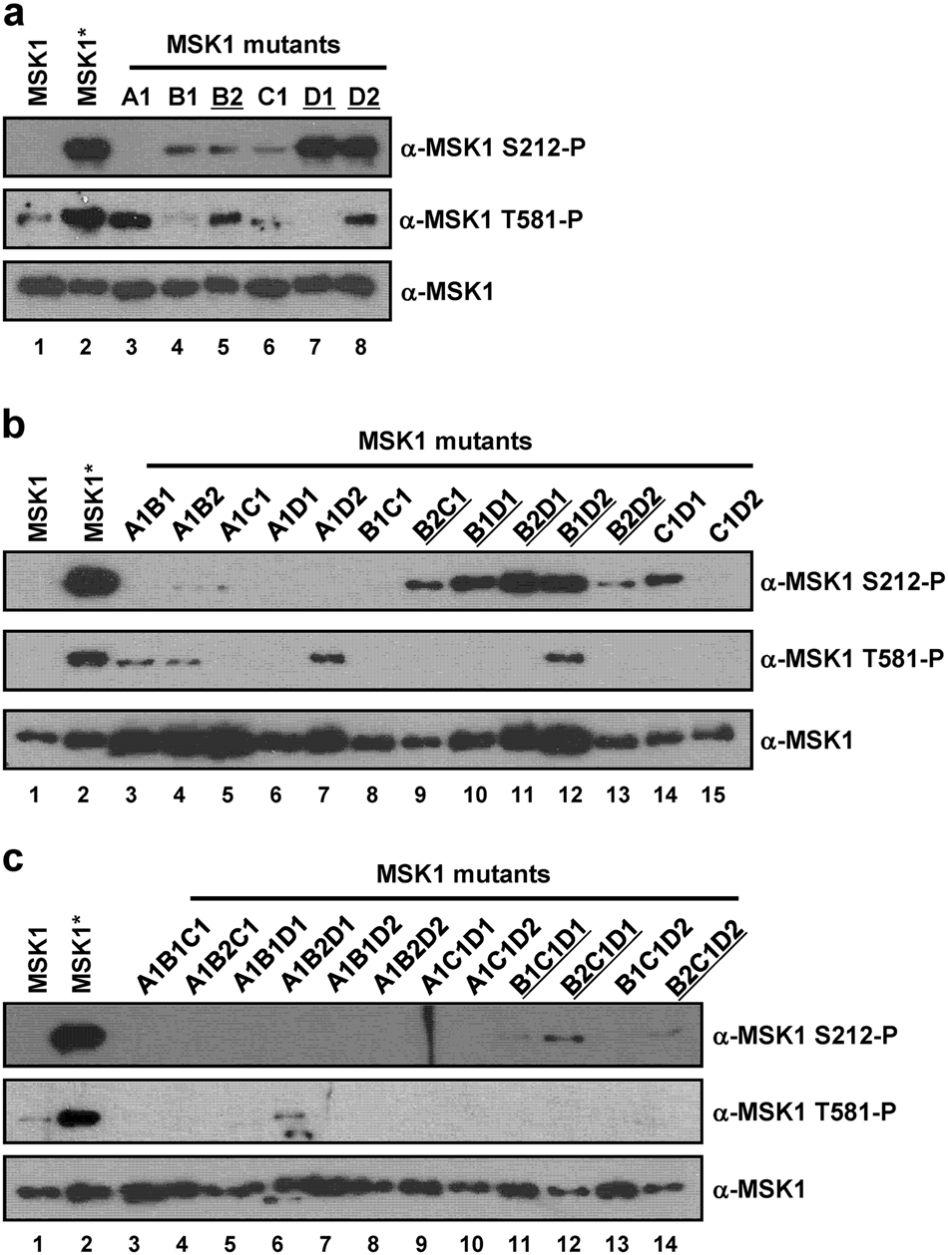
Characterization of the phosphorylation status of MSK1 phosphomimetic mutants. **a**–**c** The phosphorylation status in the activation loops of purified wild-type and phosphomimetic MSK1 with a single (**a**), double (**b**), and triple (**c**) number of mutant fragments was monitored by immunoblotting with anti-S212 and anti-T581 phospho-specific antibodies. MSK1 and MSK1* wild-type MSK1 were prepared in the absence and presence of MKK6ca co-expression, respectively. The data are representative of at least three independent experiments. MSK1 phosphomimetic mutants with active histone kinase activity (Fig. 4) are indicated by underlining

Phosphorylation of critical residues in the activation loop is common in activation of many protein kinases, including MSK1. As negative charges in the hydrophobic motif of MSK1 led to constitutive kinase activity, we examined whether the enhanced kinase activity of MSK1 was accompanied by relevant phosphorylation events in the activation loops (S212 and T581 phosphorylation in the NKD and CKD, respectively). The purified MSK1 proteins used in Fig. 4 were subjected to immunoblot analyses using phospho-specific antibodies against phosphorylated serine 212 and threonine 581 of MSK1 (Fig. 5). Interestingly, we found that phosphorylation of serine 212 and threonine 581 in the activation loops of MSK1, both pivotal events in the activation of MSK1, were not consistently increased among mutant proteins with enhanced histone kinase activity (i.e., MSK1 mutants containing B2 fragment) (Fig. 5a–c). This is in clear contrast to wild-type MSK1*, which showed an increased level of phosphorylation in both activation loops upon MKK6ca-mediated activation (Fig. 5a–c, lane 2 vs. lane 1). Phosphorylation of serine 212, but not threonine 581, appeared to correlate with the histone kinase activity of MSK1 (Fig. 5a, b), although not without exception. For example, phosphorylation on serine 212 in the C1D1 mutant, although weak (Fig. 5b, lane 14), substantially diminished H3S10 phosphorylation activity (Fig. 4c, lane 16). In addition, serine 212 phosphorylation levels were not a clear indicator of relative kinase activity, given that mutants with barely detectable phosphorylation on serine 212 (Fig. 5c, lanes 12 and 14) showed significant H3S10 phosphorylation activity (Fig. 4d, lanes 12 and 14). Taken together, these results suggest that S212 phosphorylation in the NKD generally correlates with enhanced MSK1 histone kinase activity, but the kinase-active mutants do not fully recapitulate the phosphorylation status of activated wild-type MSK1*.

The results of phosphomimetic mutation studies shown in Figs. 4 and 5 prompted us to investigate whether constitutively active MSK1 mutants with varying degrees of phosphorylation exhibit enhanced coactivator function in p53-dependent *p21* transcription. As glutamic acid substitutions in the hydrophobic motif appeared to activate the histone kinase activity of the NKD, B2 fragment-containing active MSK1 mutants were further characterized for their relative coactivator function (Supplementary Fig. 4). However, we found that these MSK1 mutants showed no enhanced coactivator function, suggesting that mere hyperactivation of the kinase activity is not sufficient to recapitulate upstream activation signals derived from MKK6ca. Therefore, intricate regulation of MSK1 through multiple phosphorylation events and elevated kinase activities appears to be important for the enhanced nucleosomal response in *p21* transcription.

## Discussion

Our study shows that MSK1 serves as a transcriptional coactivator of the tumor suppressor p53 through direct interaction with p53, such that activation of the p38 MAPK pathway enhances its coactivator function, resulting in a greater transcriptional response of the p53 target gene *p21*. MSK1 has been reported to induce transcriptional enhancement in concert with other transcriptional activators but, to the best of our knowledge, this is the first report demonstrating a direct connection with p53. We demonstrate that p53-dependent phosphorylation of chromatin by MSK1 enhances *p21* transcription, as evidenced by in vitro chromatin transcription as well as cell-based analyses. More importantly, it appears that the coactivator function of MSK1 is not entirely dependent on kinase activity; it is also partly affected by the proper phosphorylation status.

For MSK1 to elicit an appropriate biological response, it is crucial that it be properly recruited to the promoter region of target genes. Multiple studies have revealed that many transcriptional activators, such as Elk1, activator protein-1, NF-κB, and RARα, have a role in recruiting MSK1 to specific promoter regions for localized modification of chromatin^8,10,41,42^. However, no reports have clearly demonstrated p53-dependent recruitment of kinases to chromatin to modulate transcription. AMP-activated protein kinase (AMPK) was previously implicated in histone H2B serine 36 phosphorylation, although the role of p53 in AMPK targeting to chromatin was not clearly demonstrated^31^. One study similar to ours showed enhanced recruitment of MSK1 and increased H3S10 phosphorylation in the *p21* promoter but did not identify a mechanism to account for MSK1 activation and targeting^23^. Our findings support a novel connection between MSK1 and p53, showing that p53 has a direct role in the recruitment of MSK1 to p53-binding sites in the *p21* locus, and further warrant a future genome-wide analysis of specific MSK1 targeting and the kinetics of H3S10 and H3S28 phosphorylation on p53 target genes.

MSK1 activity is controlled by multiple phosphorylation events. Intra- and intermolecular phosphorylation in multiple functional domains have been shown to have crucial roles in MSK1 activation^4,5^. However, how phosphorylation at individual sites contributes to the regulation of MSK1 activation has not been thoroughly investigated. MSK1 shares a common activation mechanism with the kinases AGC and CaMK, reflecting the presence of highly conserved phosphorylation sites and regulatory motifs^43^. Activation of AGC kinase family members involves phosphorylation in the activation loop and hydrophobic motif. These phosphorylation events stabilize and activate AGC kinase by positioning the kinase domain in a catalytically active conformation, thereby enabling transfer of the phosphate group from ATP to substrates^44,45^. Consistent with this mechanism, our study suggests that phosphorylation of the hydrophobic motif (serine 376 and 381) is the most significant event in activation of the histone kinase activity of MSK1. Negative charges in the hydrophobic motif region appear to be sufficient to induce full activation of the NKD independently of C-terminal kinase activity.

We anticipated that the elevated kinase activity of MSK1 would fully reconstitute the coactivator function of MSK1 in the absence of an upstream activation signal. However, the lack of a robust stimulatory effect of constitutively active MSK1 in p53-dependent *p21* transcription (Supplementary Fig. 4) suggests that proper phosphorylation status of MSK1 in concert with additional factors also contributes to activation of p53 target gene transcription. This inference is supported by several lines of evidence. First, MSK1 with basal kinase activity can still modestly stimulate *p21* transcription in vitro and in cells. Second, considering that a fully phosphorylated H3-containing chromatin template only modestly increased transcription in vitro, it is likely to be that activated MSK1* is involved in other processes in addition to chromatin phosphorylation-mediated nucleosomal responses. Finally, our studies showed that phosphorylation of MSK1 and activation of its kinase activity could be uncoupled in certain phosphomimetic mutants. Despite possessing kinase activity comparable to that of wild-type MSK1*, these kinase-active phosphomimetic mutants were not effective in p53-mediated *p21* transcription, suggesting the role of multiple phosphorylation events in the coactivator function of MSK1. As MSK1 is known to associate with the MLL1 complex^46^, the BAF complex, and 14-3-3 protein^47^, proper posttranslational modifications might allow MSK1 to synergize with these transcription factors.

**Fig. 6:**
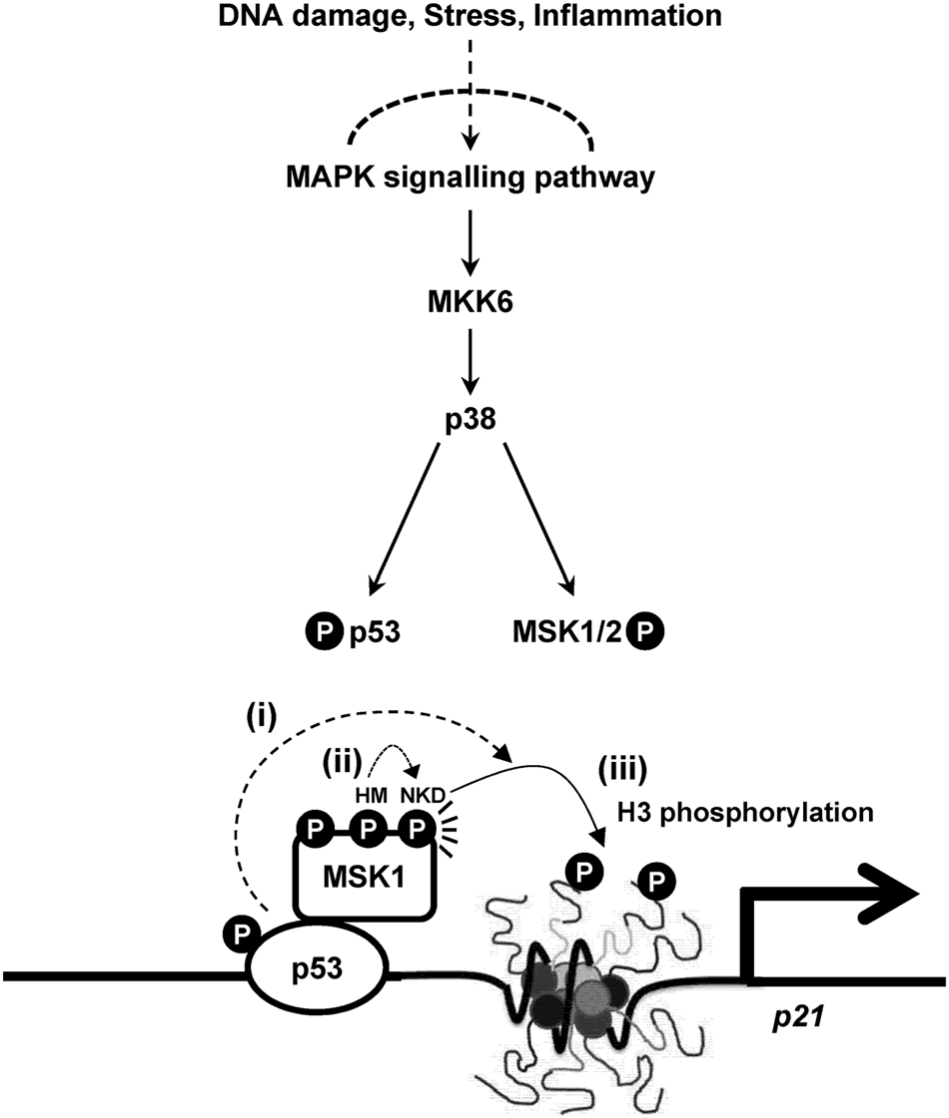
Mechanistic model of the coactivator function of MSK1 in p53-dependent *p21* transcription. p38 MAPK phosphorylates two downstream targets, p53 and MSK1/2, which act cooperatively to stimulate transcription of the p53-responsive *p21* gene as follows: (i) p38 phosphorylates p53 and increases its protein stability and transcription activity; (ii) p38-dependent phosphorylation triggers autophosphorylation of the NKD, which is stimulated by negative charges acquired by phosphorylation events in the hydrophobic motif (HM) and in turn activates the histone kinase activity of MSK1; (iii) activated MSK1 phosphorylates nucleosome phosphorylation through direct interactions with p53, which increases *p21* transcription

Various stress signals turn on MAPK signaling pathways; among these, activation of p38 MAPK is critical for stimulating p53-responsive genes (Fig. 6). p38 MAPK is a potential tumor suppressor, acting through p53, to elicit tumor suppression by intervening in apoptotic responses and cell cycle control processes^48^. Our results show that simultaneous activation of p53 and MSK1 by p38 MAPK, and their cooperation are also required for efficient p53 target gene expression. MSK1 appears to be a more appropriate druggable target than p38 MAPK, owing to its substrate selectivity. Specific inhibitors of MSK1 kinase activity have been shown to selectively suppress NF-κB target gene transcription and are thus considered to be potential therapeutics for treating abnormal inflammation^8^. Given that a synthetic peptide of the hydrophobic motif of the AGC protein kinase family is known to mimic intramolecular MSK1 activation^43^, enhancing MSK1 function without disturbing upstream kinase signaling pathways is an approach that should be further explored as a potential tumor suppression strategy.

## Electronic supplementary material

Supplementary Information
