# Supplementary material for: MSK1 functions as a transcriptional coactivator of p53 in the regulation of p21 gene expression
Source: Exp Mol Med. 2018 Oct 10;50(10):132. doi: 10.1038/s12276-018-0160-8 (PMC6180136; doi:10.1038/s12276-018-0160-8)
Supplement: Supplementary file 1 — Supplementary Information [file 12276_2018_160_MOESM1_ESM.pdf]

SUPPLEMENTARY FIGURES

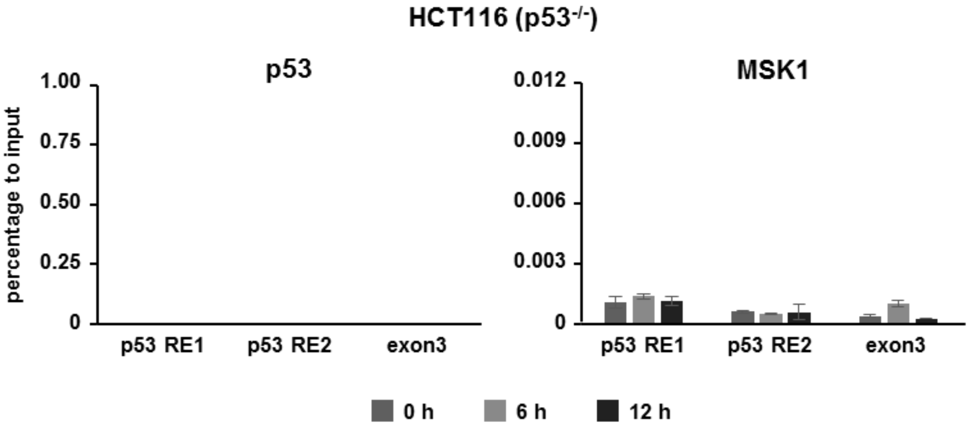

Supplementary Figure 1. ChIP analyses of p53 and MSK1 on the *p21* locus during p53-dependent transcription in p53-null HCT116 cells.

p53-deficient HCT116 colorectal cancer cells were treated with doxorubicin for the indicated times and ChIP analyses were performed with indicated antibodies. Error bars indicate standard deviations from three independent ChIP analyses.

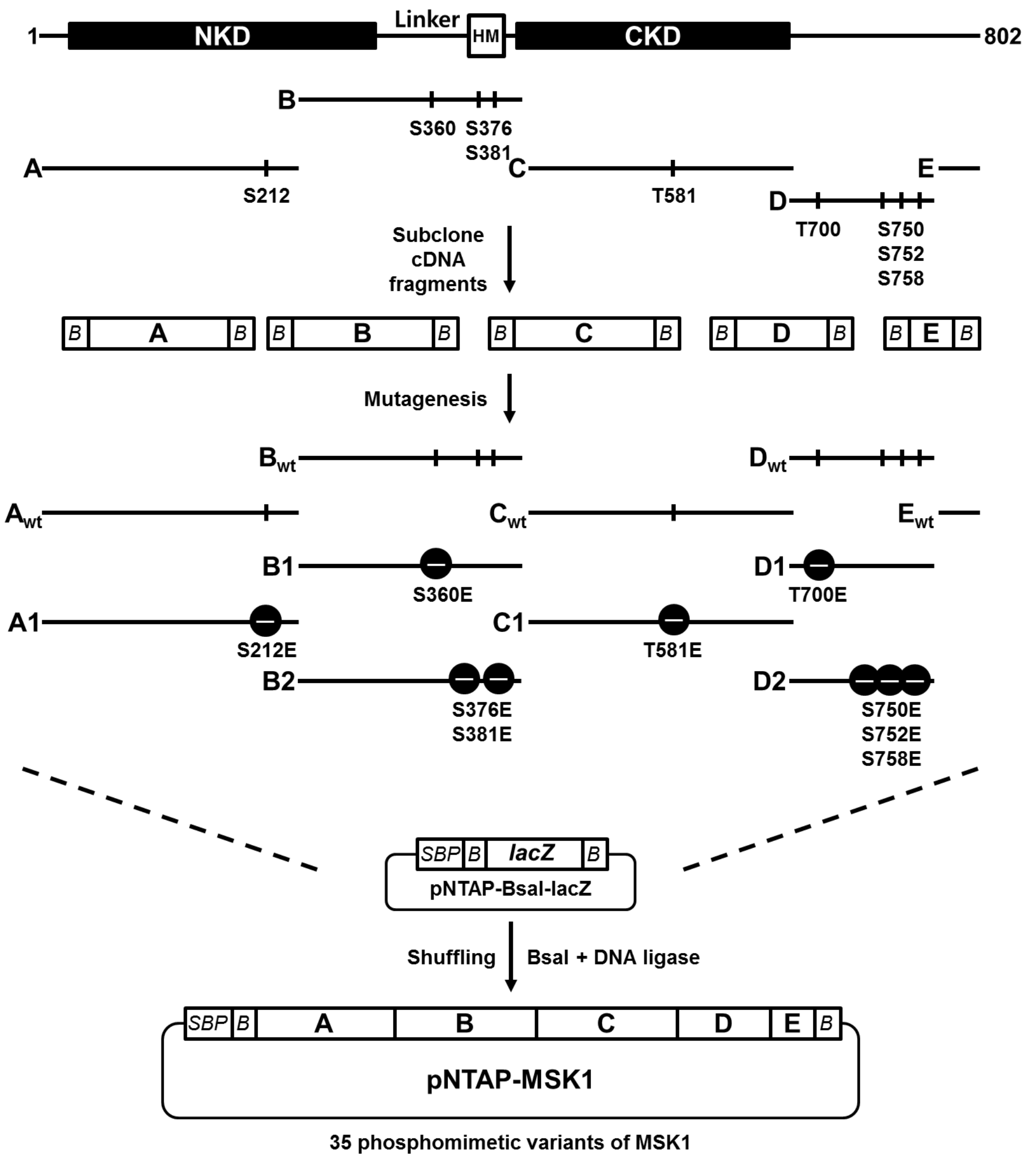

Supplementary Figure 2. Overview of MSK1 phosphomimetic mutants generation.
Five MSK1 cDNA fragments were subcloned into a pUC19-derived entry vector. Primers were designed to introduce *Bsa*I recognition sites to the 5' and 3' end of each MSK1 fragment in reverse orientation so that *Bsa*I recognition sites can be removed upon cDNA assembly and that resulting complimentary overhang sequences can be joined in a specific order. The entry vectors were used as templates for generation of phosphomimetic mutations from serine/threonine to glutamic acid by site-directed mutagenesis. For the assembly reaction, the pNTAP-*Bsa*I-LacZ vector was digested with *Bsa*I to release the *lacZ* cassette (whose 5' and 3' ends were flanked by *Bsa*I recognition sites in reverse orientation) and MSK1 entry clones were then subcloned by ligation of compatible overhangs. *B*, *BsaI* recognition site; SBP, streptavidin-binding peptide.

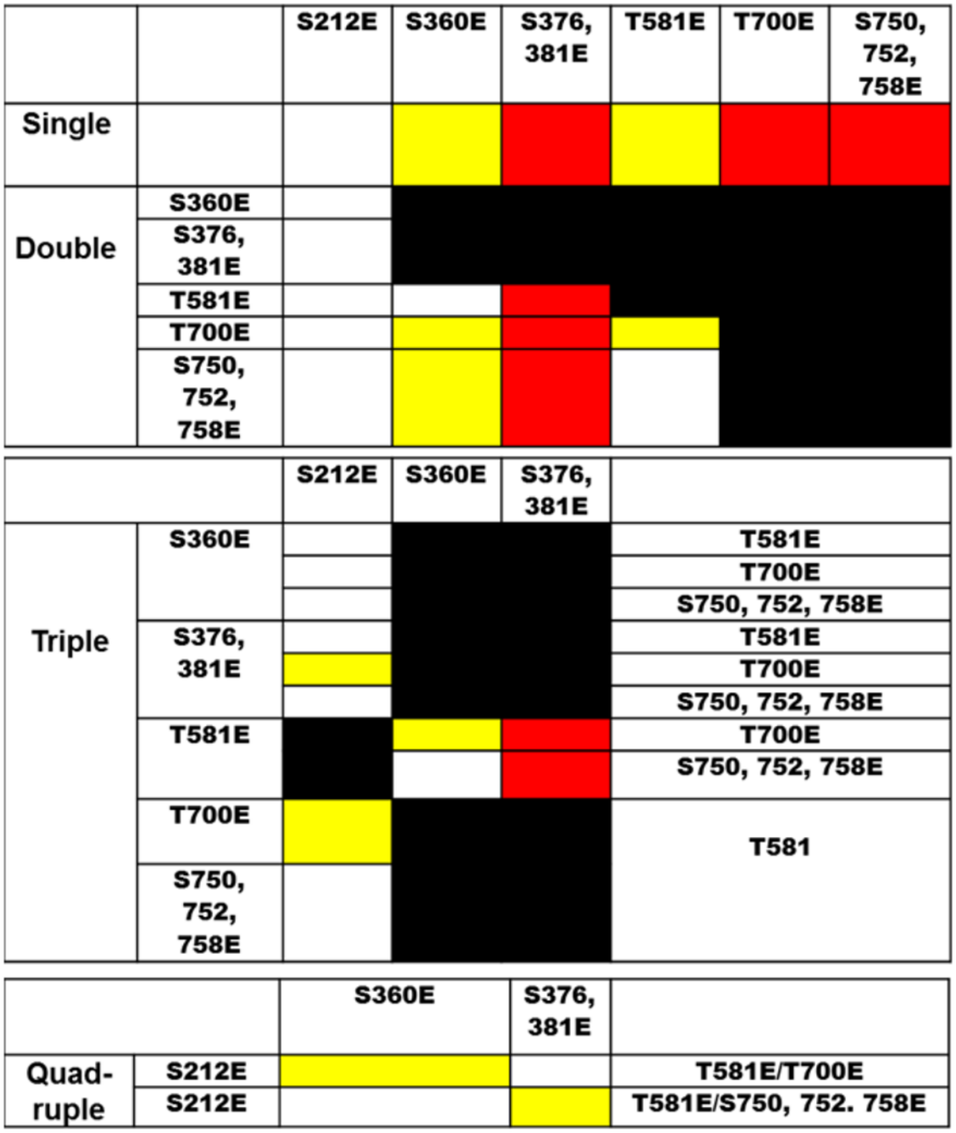

Supplementary Figure 3. Graphical depiction of the relative histone kinase activity of 35 MSK1 phosphomimetic mutants.

H3S10 phosphorylation activities of MSK1 phosphomimetic mutants relative to MSK1* are grouped into three classes marked by white (inactive), yellow (modestly active) and red (highly active).

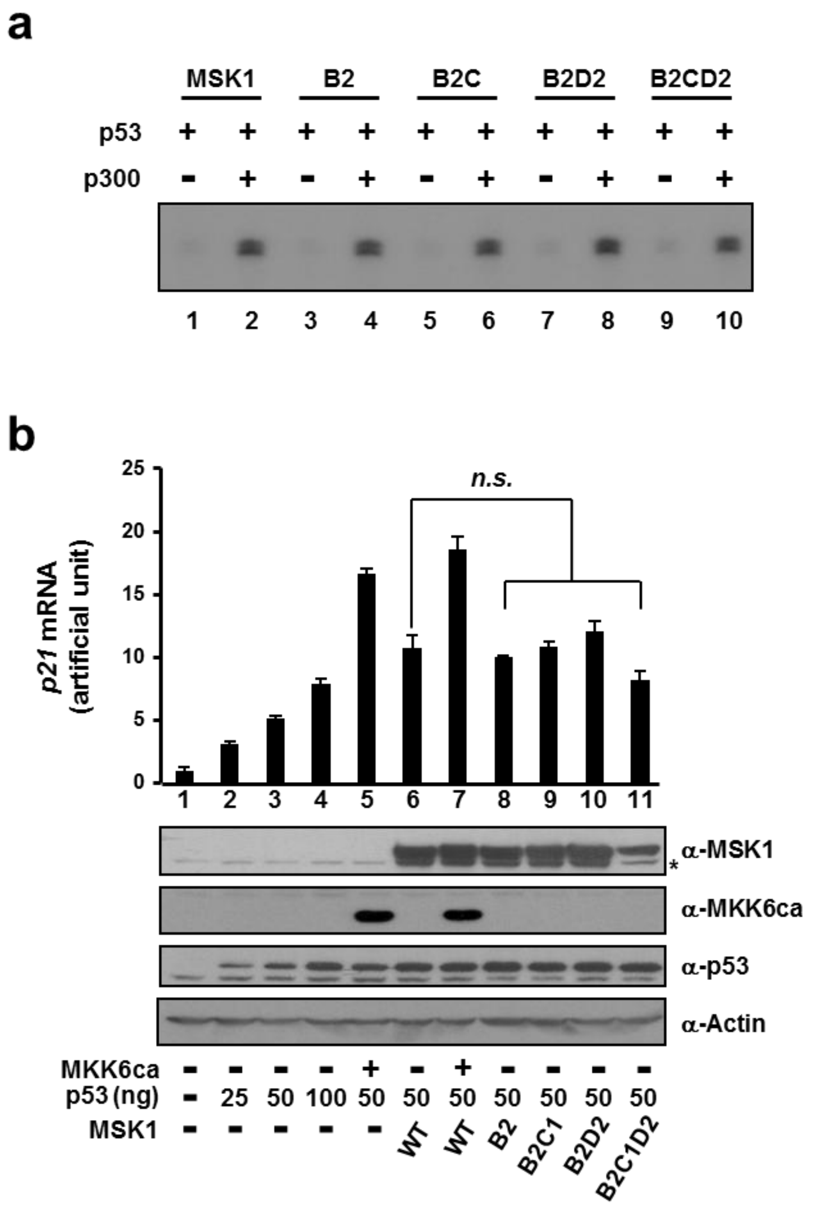

Supplementary Figure 4. Characterization of constitutively active (B2 fragment-containing) MSK1 phosphomimetic mutants in p53-depenedent *p21* expression.

(**a**) Effects of constitutively active MSK1 phosphomimetic mutant proteins in p53-mediated *in vitro* transcription of a chromatin template. Standard p53- and p300-dependent transcription from chromatinized p53ML plasmid was performed. Reactions contained 15 ng of indicated MSK1.

(**b**) Effects of constitutively active MSK1 phosphomimetic mutants in endogenous *p21* transcription. H1299 cells were transfected with combination of plasmids expressing p53, MKK6ca, MSK1 wild type and phosphomimetic mutants as indicated. Total RNA and cell lysates were prepared and analyzed for *p21* mRNA (top panel) and for protein levels by immunoblotting with indicated antibodies (bottom panels). Error bars represent mean standard deviation from three triplicate samples. The significance of the differences in *p21* expression was evaluated by Student's t-test (*n.s.,* not significant). Asterisk indicates endogenous MSK1.

SUPPLEMENTARY TABLES

Supplementary Table 1. Oligonucleotide sequences for generation of five MSK1 cDNA fragments.

Fragments	Amino acid position	Forward Primer	Reverse Primer	5' overhangs	3' overhangs
A	1-227	*GGTCTC*AAATGGAGGAAGAAGGTGGCAGCA	*GGTCTC*CCCTCTGACAATATCTGGTGCCA	AATG	CCTC
B	228-428	*GGTCTC*AGAGGGGGAGATTCAGGACATGA	*GGTCTC*CTTCAAATCTAGGTCATAGTGTTGATA	GAGG	TTCA
C	429-690	*GGTCTC*TTGAAGGACAAACCCCTGGGAGAAGGT	*GGTCTC*TCCATCTTGTAGCCATTCATTGTACCTCA	TGAA	CCAT
D	691-792	*GGTCTC*GATGGAAGTCAGCTGTCCTCCAATGGA	*GGTCTC*CGTCTCCGGGTTATTGCTGTCGGCAGGA	ATGG	GTCT
E	793-802	*GGTCTC*GAGACGCTCTTCCAGTTCTCGGACTCAGT	*GGTCTC*GAAGCCATAGAGCCCACCGCATCCCCA	AGAC	CCAT

Sequences in italic indicate Bsa1 restriction sites.

Supplementary Table 2. Oligonucleotide sequences for introduction of glutamic acid mutation into MSK1 fragments.

Fragment	Mutation site	Primer name	Primer sequence
A1	S212E	S212E_FOR	GCATAT*GAG*TTTTGTGGAACTATTGAATACATGG
		S212E_REV	ACAAAA*CTC*ATATGCTCTTTCAGTTTCATCAGC
B1	S360E	S360E_FOR	ACTTAT*GAG*CCCGCAGCCCTGCCCCAGAGTT
		S360E_REV	TGCGGG*CTC*ATAAGTGGGGTCCATTTCTGTGAACT
B2	S376E/S381E	S376E S381E_FOR	*GAG*TTTGTTGCTCCT*GAG*ATCCTATTCAAGCGTAATGCAGCT
		S376E &S381E_REV	*CTC*AGGAGCAACAAA*CTC*ATAGCCCTGAAACAGCTTCTCAGA
C1	T581E	T581E_FOR	CTGAAG*GAG*CCATGCTTCACCCTTCATTATGCC
		T581E_REV	GCATGG*CTC*CTTCAGGGGCTGATTATCCGGTG
D1	T700E	T700E_FOR	CTGATG*GAG*CCGGATATTCTAGGATCTTCCGGA
		T700E_REV	ATCCGG*CTC*CATCAGAGGATTGGAGGACAGCTG
D2	S750E/S752E /S758E	S750E, S752E, & S758E_FOR	*GAG*ACC*GAG*ACCGAGACGCGCAGC*GAG*TCCAGTGAGAGTTCCCATTCTTCTT
		S750E, S752E, & S758E_REV	*CTC*GCTGCGCGTCTCGGT*CTC*GGT*CTC*AGTCTTTTTCATTTTTCTTCTCTTAGCC

Sequences in italic indicate codon sequences of glutamic acid. FOR, forward primer; REV, reverse primer.
